# Utilization of the partograph and its associated factors among obstetric care providers in the Eastern zone of Tigray, Northern Ethiopia, 2017: a cross-sectional study

**DOI:** 10.11604/pamj.2019.34.181.18246

**Published:** 2019-12-06

**Authors:** Guesh Welu Gebreslassie, Desta Abraha Weldegeorges, Natneal Etsay Assefa, Berhanu Gebresilassie Gebrehiwot, Senait Gebreslasie Gebremeskel, Betell Berhane Tafere, Gdiom Gebreheat, Tesfay Tsegay Gebru, Dessalegn Kiros, Kidanemaryam Berhe Tekola, Tsehaynesh Gidey Welesamuel

**Affiliations:** 1 Department of Midwifery, College of Medicine and Health Sciences, Aksum University, Aksum, Ethiopia; 2 Department of Midwifery, College of Medicine and Health Sciences, Adigrat University, Adigrat, Ethiopia; 3 Departments of Nursing, College of Medicine and Health Sciences, Adigrat University, Adigrat, Ethiopia; 4 Department of Nutrition and Dietetics, School of Public Health, College of Health Sciences, Mekelle University, Mekelle, Ethiopia; 5 Department of Public Health, College of Medicine and Health Sciences, Aksum University, Aksum, Ethiopia

**Keywords:** Ethiopia, obstetric care providers, partograph, utilization

## Abstract

**Introduction:**

the partograph is a pre-printed paper form used in monitoring the progress labor. It was initially introduced by Philpot; and endorsed by the World Health Organization as a simple and accurate instrument for early recognition of complications of labor. Our study was conducted to evaluate the utilization of the partograph and associated factors among obstetric care providers in the Eastern zone of Tigray, Northern Ethiopia 2017.

**Methods:**

a cross-sectional study was conducted in the Eastern zone of Tigray. Four hundred and fourteen participants were randomly selected from the Eastern zone weredas (districts). Data were collected using a self-administered questionnaire. The data were entered into epi data version 3.5 and exported to SPSS V-20 for analysis. Bivariate and multivariate analysis were done to determine the association between a dependent variable and independent variables at P-value <0.05.

**Results:**

of the 406 obstetric care providers, 83% of them had utilized the partograph to monitor labor. In addition, utilization of the partograph were statistically associated with being female (AOR=2.09, 95%CI= (1.11, 3.93), age group of 20-25 (AOR=0.25, 95%CI= (0.07, 0.88), being a diplomat midwives (AOR=0.01, 95%CI= (0.00, 0.28)) and having qualified from pre-service training (AOR=0.01, 95%CI= (0.02, 0.05)).

**Conclusion:**

participants’ utilization of the partograph was generally good. However, most of them were using it incorrectly. Age, gender, level of educational, year of qualification from pre-service training were the variables that showed association with the utilization of the partograph. The provision of on-the-job training on the partograph is recommended to improve partograph utilization.

## Introduction

The partograph is an easy tool designed to provide a continuous pictorial overview of labor and has been shown to improve the outcomes when used to monitor labor. It is a single sheet of paper which includes information about the fetal condition, maternal condition and labor progress [1]. It is a practical method when employed in a busy labor room with many cases, but limited personnel to screen for abnormal labor. With its use, there is no need to record labor events repeatedly. It helps predict deviation from normal progress of labor and supports timely and proven intervention. It also helps to show any deviation from the normal to the person conducting labor [1,2]. Prolonged and obstructed labor accounts for 8%-10% of maternal deaths and mechanical obstruction in the second stage is a possible complication in about 1%-2% of labors [2]. Ethiopia is still one of those developing countries with an estimated maternal mortality ratio of 420/100,000 in 2013. World Health Organization recommends using the partograph to monitor labor and delivery, with the objective of improving health care and reducing maternal and fetal morbidity and mortality [1,3].

According to a study done by WHO in South-East Asia, the partograph was the necessary tool in the management of labor and its findings indicated that appropriate utilization reduced prolonged labor (from 6.4% to 3.4%), the proportion of labor requiring augmentation (from 20.7% to 9.1%), emergency cesarean section (from 9.9% to 8.3%) and stillbirths (from 0.5% to 0.3%) [1,4]. Although the partograph is a simple and inexpensive tool that prevents maternal deaths and complications due to obstructed or prolonged labor, it is not as widely implemented as it should be. Studies done in Nigeria reported that only 25% to 33% of caregivers surveyed were using the partograph for routine monitoring of labor [3,5]. Caregivers may resist using the tool if they have insufficient knowledge and do not fully understand why they have been asked to use the tool. Non-availability of preprinted partograph has also been reported as a cause for non-utilization [3]. Filling the partograph is also seen as an additional task for a busy health worker in such a situation and may not be motivated to complete the partograph. However, the challenges to the implementation of the partograph, including insufficient knowledge, non-availability of preprinted partograph and workload pressure, can all be addressed with further education on the purpose of the partograph and local managerial support [3].

According to a study done in Addis Ababa, 57.4% of obstetric caregivers used the modified WHO partograph to monitor women in labor in public health institutions. Among those who used partograph, 80.4% used it routinely while 8.9% and 10.7% of them have used it occasionally and sometimes respectively [6]. According to a study in African countries, the utilization of the partograph is poor despite preparing the tool that is simple and inexpensive for intrapartum monitoring of labor [5,7]. Similarly, a study in Ethiopia revealed low utilization of the partograph [6]. The lack of preprinted partograph in the health institutions, being a general practitioner, poor knowledge and attitude towards partograph were the reason for not using partograph during labor; however, all these challenges to the use of the partograph can be resolved by provisions of pre-service and on the job training on partograph [6]. However, little is known about the utilization of partograph among obstetric care providers; Therefore, the objective of this study was to find the utilization of the partograph and associated factors among obstetric care providers in the eastern zone of Tigray, Northern Ethiopia.

## Methods

An institutional based cross-sectional study design was conducted to assess the utilization of the partograph and its associated factors among obstetric care providers in the eastern zone, Northern Ethiopia from December 1^st^, 2016 to February 1^st^, 2017. The sample size was calculated using a single population proportion formula and the following assumptions were considered: proportion of previous available study on utilization of partograph 57.3% (p=0.573) [6], level of significance to be 5% (α = 0.05), 95% confidence level (Zα/2 = 1.96) and absolute precision or margin of error to be 5% (d = 0.05). Based on this, adding a 10% non-responsive rate, the final sample size required for this study were 414 health care providers. The sample size was proportionally allocated to the size of each of the seven weredas/districts and two town administration then participants from each wereda/district were randomly selected using a simple random sampling method. An interviewer semi-structured questionnaire was used for data collection. Different relevant literatures were reviewed to develop the tool and the tool was pretested on 10% of the sample size on similar study participants in Teklisiwuat primary hospital. The collected questionnaire was checked for completeness, coded and entered into SPSS statistical package for analysis. Descriptive data were presented using text and table. Both bivariate and multivariate logistic regression analyses were conducted. Variables with a significant level of (p<0.2) in bivariate analysis were entered into multivariate logistic regression. Odds ratio with 95% confidence intervals were computed to identify the presence and strength of association, and statistical significance was declared if p<0.05. Ethical clearance and approval were obtained from Adigrat university research and community service director with the code number of AGU/CMHS/029/08. An official letter of cooperation was obtained from Tigray regional health bureau, the eastern zone administration and from each wereda/district health office. Verbal consent from the medical directors of each institution was obtained. Those respondents who were not willing to participate in the study were not forced to be involved. They were also informed that all data obtained from them will be kept confidential by using codes instead of any personal identifiers and is meant only for the purpose of the study.

**Ethics approval:** ethical clearance and approval was obtained from Adigrat university research and community service director with the protocol number of AGU/CMHS/029/08, and oral consent was taken from the study participants.

## Results

**Socio-demographic characteristics of the study participants:** a total of 406 participants were enrolled in the study making a response rate of 98.1%, of these 211(52%) were females. Two hundred and five (50.5%) participants were enrolled from health centers and the rest from hospitals. All of the participants were between the age of 20 and 41 years old with a mean and standard deviation of 27.09 and 4.26 respectively. Three hundred and forty (83.7%) of the respondents were Orthodox Christian followed by Muslims (9.1%). Three hundred and thirty-seven (83.0%) of the respondents have ever practiced the partograph. Most of the respondents were on the age group of 20-25. Most of the participants level of education 138(34.0%) were diploma midwife and 85(20.9%) of the respondents were diploma nurses. One hundred and fifty (36.9%) of the respondents were qualified from their pre-service training in the last year followed by three years ago (20.9%). Two hundred and sixty-five (65.3%) of the respondents take in-service training (Table 1).

**Table 1:** socio-demographic characteristics of obstetric care providers in the eastern zone of Tigray, Northern Ethiopia, 2017

Variable		Frequency(N)	Percentage (%)
**Gender**	Male	196	48
	Female	211	52
**Age**	[20-25]	183	45.1
	[25-30]	147	36.2
	[30-35]	61	15.0
	>35	15	3.7
**Religion**	Orthodox	340	83.7
	Muslim	37	9.1
	Catholic	29	7.1
**Level of education**	Midwife (diploma)	138	34.0
	Midwife (degree)	73	18.0
	Nurse(diploma)	85	20.9
	Nurse (degree)	53	13.1
	Health officer	47	11.6
	Medical doctor	10	2.5
**Place of work**	Hospital	201	49.5
	Health center	205	50.5
**Unit/ward currently working**	ANC	83	20.4
	FP	104	25.6
	Labor ward	176	43.3
	other unit	43	10.6
**In-service training has taken**	yes	265	65.3
	No	141	34.7
**A qualification from pre-service training**	This year	150	36.9
	Two years ago	78	19.2
	Three years ago	85	20.9
	Four years ago	14	3.4
	Before five years	79	19.5
**Years of experience**	< 1 yea r	176	43.3
	1-5 years	162	39.9
	5-10 years	47	11.6
	> 10 years	21	5.2

**Utilization of partograph among obstetric health care providers:** the prevalence of utilization of partograph among obstetric care providers in the eastern zone was (83%). One hundred and sixty four (40.4%) of the respondents considered partograph as a chart for monitoring of labor by doctors only while 242(59.6%) considered it as a chart for monitoring of labor by all health professionals and 339(83.5%) of the respondents considered partograph as a chart for monitoring of labor by a midwife only. Three hundred and sixty-seven (90.4%) and 373(91.9%) of the respondents indicated that utilization of partograph reduces maternal and newborn death respectively. Of the participants, 33(8.1%) and 39(9.6%) indicated that the partograph couldn’t reduce maternal and newborn mortality respectively. Three hundred and twenty-nine (81.0%) of the respondents were familiar with the alert line and they considered in normal progress of labor, the graph should fall on the left of the alert line whereas 77(19.0%) of them don't know about the alert line. About 43% of the respondents indicated that in the normal progress of labor, the graph should fall on the right of the alert line and it didn't have any problem if it didn't cross the action line. A majority (92.4%) of the participants indicated that the progress of labor is assessed by the degree of cervical dilation and descent of the fetal part. Three hundred (73.9%) of the participants usually record maternal and fetal information on to the partograph and 26.1% didn’t record. Of the participants, 192(47.3%) record the information during diagnosis of labor, 116(28.6%) during the active first stage of labor and 20(4.9%) after delivery of the fetus. Majority of the participants (84.5%) have available partograph chart on the labor ward. Around 96.1% of the participants used the partograph to monitor patients during labor and 318 (78.3%) of them used it routinely and 72(17.7%) rarely. Three hundred and forty one (84.0%) of the participants used the partograph to monitor for every woman and 222(54.7%) used once/30 minutes (Table 2).

**Table 2:** bivariate and multivariate regression analysis on utilization of partograph associated factors among obstetric health care providers in the eastern zone of Tigray Northern Ethiopia, 2017

Variables		General utilization, N (%)		COR(95% CI)	AOR(95% CI)
		Yes (%)	No (%)		
**Gender**	Male	164(84.1%)	31(15.9%)	1	1
	Female	173(82.0%)	38(18.0%)	1.16(0.69, 1.96)	2.09(1.11, 3.93)*
**Age**	[20-25]	135(40.1%)	48(69.6%)	1	1
	[25-30]	134(39.8%)	13(18.8%)	0.47(0.31, 0.70)*	0.25(0.07, 0.88)*
	[30-35]	53(15.7%)	8(11.6%)	-	
	>35	15(4.5%)	0(0%)	-	
**Level of education**	Midwife (diploma)	133(39.5%)	5(7.2%)	1	1
	Midwife (degree)	63(18.7%)	10(14.5%)	0.09(0.02, 0.44)*	0.01(0.00, 0.05)*
	Nurse(diploma)	52(15.4%)	33(47.8%)	0.37(0.08, 1.67)	0.19(0.02, 1.73)
	Nurse (degree)	36(10.7%)	17(24.6%)	1.48(0.36, 6.13)	0.54(0.06, 4.78)
	Health officer	46(13.6%)	1(1.4%)	1.10(0.25, 4.79)	0.12(0.01, 1.35)
	Medical doctor	7(2.1%)	3(4.3%)	0.05(0.01, 0.56)*	0.01(0.00, 0.28)*
**Place of work**	Hospital	175(51.9%)	162(48.1%)	1	1
	Health center	26(37.7%)	43(62.3%)	0.56(0.33, 0.95)*	0.41(0.15, 1.12)
**In-service training taken**	Yes	226(67.1%)	111(32.9%)	1	1
	No	39(56.5%)	30(43.5%)	0.69(0.38, 1.08)	0.85(0.43, 1.66)
**Unit/ward currently working**	ANC	70(20.8%)	13(18.8%)	1	1
	FP	74(22.0%)	30(43.5%)	0.96(0.35, 2.60)	22.45(2.01, 250.34)
	Labor ward	157(46.6%)	19(27.5%)	2.09(0.84, 5.20)	67.34(6.73, 673.94)
	other unit	36(10.7%)	7(10.1%)	0.62(0.24, 1.59)	10.85(1.09, 107.80)
**Religion**	Orthodox	280(83.1%)	60(87.0%)	1	-
	Muslim	30(8.9%)	7(10.1%)	2.89(0.67, 12.50)	-
	Catholic	27(8.0%)	2(2.9%)	3.15(0.60, 16.49)	-
**Qualification from pre-service training**	This year	140(41.5%)	10(14.5%)	1	1
	Two years ago	56(16.6%)	22(31.9%)	2.75(0.59, 12.87)	2.50(0.36, 17.63)
	Three years ago	50(14.8%)	35(50.7%)	15.12(3.42, 66.97)*	-
	Four years ago	14(4.2%)	0(0.0%)	-	
	Before five years	77(22.8%)	2(2.9%)	-	
**Years of experience**	< 1 yea r	156(46.3%)	20(29.0%)	1	-
	1-5 years	114(33.8%)	48(69.6%)	1.27(0.26, 6.42)	-
	5-10 years	46(13.6%)	1(1.4%)	0.91(0.19, 4.32)	-
	> 10 years	21(6.2%)	0(0.0%)	0.59(0.17, 2.78)	

* Statistical significance (p<0.05), 1= Reference, COR = Crude odds ratio, AOR= Adjusted odds ratio

**Factors associated with partograph utilization among obstetric health care providers:** in Table 2 of the bivariate logistic regression analysis, overall partograph utilization was significantly associated with socio-demographic characteristics like age, profession/level of educational, place of work/type of institution and year of qualification from pre-service training i.e. age group of 25-30 were less likely to utilize partograph than age group of 20-25 (p≤0.001, COR=0.47, 95% CI= (0.31, 0.70)), being a medical doctors and degree midwives were less likely to apply partograph than diploma midwives (p=0.015, COR=0.05, 95% CI= (0.005, 0.558)) and (p≤0.001, COR=0.09, 95% CI= (0.02, 0.44)) respectively. Those who were working in the health center were less likely to apply the partograph (p=0.032, COR= 95% CI= (0.33, 0.95)) than those who were working in the hospital. Health professionals who were qualified before three and before four years were more likely to utilize the partograph than those qualified this year (p≤0.001, COR=15.12, 95% CI= (3.42, 66.97) p≤0.001, COR= 26.9, 95% CI= (6.20, 117.07)) respectively (Table 2). In the multivariate logistic regression analysis, a comparison of the utilization of partograph was significantly associated with variables like age, gender, profession/level of education and year of qualification from pre-service training (Table 2).

## Discussion

Measurement of the utilization of partograph brings health professional preferences into quality practice. Assessing the utilization of partograph was an important component for the quality of practice and to decrease maternal and neonatal death. Effective use of the partograph according to the World Health Organization has been noted to effectively reduce maternal morbidity and mortality [1]. Obstetric professionals all over the world have been encouraged to take the responsibility of ensuring effective use of the partograph to combat the maternal and neonatal morbidity and mortality [1,2]. According to our study, the overall level of utilization of partograph was 83%. This finding is higher as compared to studies conducted in North Shoa zone, Central Ethiopia (40.2%) [8], Addis Ababa (57%) [6], South Africa (64%) [9], Gambia (78%) [10] and Amhara region, Ethiopia (80%) [11]. This difference may be as a result of the time difference. However, it was lower than the studies done in Benin (98%) [12], the Niger Delta of Nigeria (98.8%)[5]. Two hundred sixty-three (64.8%) of the respondents had received job training on the partograph. This is low as compared to other studies [8,11].

This study showed that the partograph was recorded frequently by staffs working in health centers than staffs working in hospitals (50.5% vs 49.5%), supported by the findings from Addis Ababa, Ethiopia, utilization of the partograph was significantly higher among obstetric caregivers working in health centers (67.9%) compared to those working in hospitals (34.4%) [6]. However, it was in contradiction with a study done in Nigeria [13]. The result showed that there is a significant association between gender and utilization of the partograph. The odds of utilization of the partograph were two times higher among females than males (p=0.023, AOR=2.09, 95% CI= (1.109, 3.931) (Table 2)). This could be explained by the fact that female were closer to obstetric information as they have a tendency to become midwives than male. This finding is consistent with a previous study done in Amhara region, Ethiopia [11]. Being a medical doctor and being a degree midwife were 88% and 96% less likely to monitor labor using the partograph than diploma midwives (p=0.006, AOR=0.01, 95% CI= (0.00, 0.28)) and (p≤0.001, AOR=0.01, 95% CI= (0.02, 0.05)) respectively (Table 2). This is comparable with a study done in North Shoa Zone, Central Ethiopia and nineteen Ethiopian hospitals [8,14]. This might be due to the fact that medical doctors and degree midwives might use history and physical examination for diagnosis than diploma midwives.

## Conclusion

The participants' utilization of the partograph was generally good. However, most of them were used incorrectly. Age, gender, profession/level of education were the variables that affected the utilization of partograph. Provision of on-the-job training on the partograph is recommended to stakeholders and woreda health departments to improve the knowledge and attitude of obstetric care providers towards the partograph utilization and to apply appropriately. The obstetric care providers should monitor all laboring mothers with partograph, rather than other monitoring tools that lack the parameters of the partograph.

### What is known about this topic

- Appropriate utilization of partograph can reduce prolonged labor (from 6.4% to 3.4%), the proportion of labor requiring augmentation (from 20.7% to 9.1%), emergency cesarean section (from 9.9% to 8.3%) and stillbirths (from 0.5% to 0.3%);
- About 57.4% of obstetric caregivers in Addis Ababa were used the modified WHO Partograph to monitor labor in public health institutions;
- The overall prevalence of partograph utilization was low as compared to our finding.

### What this study adds

- The overall prevalence was unknown in the eastern zone Tigray and this study identified the prevalence which is 83%;
- The higher the level of education, the lower the utilization of the partograph.

## Competing interests

The authors declare no competing interests.
